# Burden of Disease Due to Air Pollution in Afghanistan—Results from the Global Burden of Disease Study 2019

**DOI:** 10.3390/ijerph21020197

**Published:** 2024-02-08

**Authors:** Omar Hahad

**Affiliations:** 1 Department of Cardiology I, University Medical Center, Johannes Gutenberg University, 55131 Mainz, Germany; omar.hahad@unimedizin-mainz.de; 2 German Center for Cardiovascular Research (DZHK), Partner Site Rhine-Main, 55131 Mainz, Germany

**Keywords:** air pollution, Afghanistan, burden of disease, risk factor, death, Global Burden of Disease Study 2019

## Abstract

Introduction: Air pollution is a significant risk factor for a range of diseases and leads to substantial disease burden and deaths worldwide. This study aimed to investigate the burden of disease in Afghanistan attributed to air pollution in 2019. Methods: Data from the Global Burden of Disease (GBD) Study 2019 were used to investigate disability-adjusted life-years (DALYs), years of life lost (YLLs), years lived with disability (YLDs), and deaths attributed to air pollution in Afghanistan. Results: In 2019, air pollution in Afghanistan was associated with significant health impacts, and contributed to 37,033 deaths (14.72% of total deaths), 1,849,170 DALYs (10.80% of total DALYs), 76,858 YLDs (2.07% of total YLDs), and 1,772,311 YLLs (13.23% of total YLLs). The analysis further revealed that lower respiratory infections, neonatal disorders, ischemic heart disease, stroke, chronic obstructive pulmonary disease, lung cancer, and diabetes mellitus were the leading causes of mortality and disease burden associated with air pollution in Afghanistan from 1990 to 2019. Comparative assessments between 1990 and 2019 underscored air pollution as a consistent prominent risk factor that ranked closely with other risk factors, like malnutrition, high blood pressure, and dietary risks, in contributing to deaths, DALYs, YLDs, and YLLs. In a comparative country analysis for the year 2019, Afghanistan emerged as having a substantial burden of disease due to air pollution, closely mirroring other high-burden nations like China, India, Pakistan, and Bangladesh. Discussion: Air pollution is one of the major health risk factors that significantly contribute to the burden of disease in Afghanistan, which emphasizes the urgent need for targeted interventions to address this substantial public health threat.

## 1. Introduction

Air pollution, as defined by the World Health Organization (WHO) as the “contamination of the indoor or outdoor environment by any chemical, physical, or biological agent that alters the natural characteristics of the atmosphere,” poses an important challenge to global public health [1]. Air pollution, characterized as a diverse mix of particles and gases, results from a combination of anthropogenically generated pollutants and those originating from natural sources. Human activities, such as industrial processes and the combustion of fossil fuels, significantly contribute to the release of pollutants into the atmosphere. Additionally, natural events, such as wildfires, volcanic eruptions, and dust storms, can introduce particles and gases that further contribute to the complex composition of air pollution. Fossil fuel combustion, especially from transportation, releases nitrogen oxides (NO_x_), including nitrogen dioxide (NO_2_), and carbon monoxide (CO). Sulfur dioxide (SO_2_) results from fossil fuel combustion for heating and power, and ozone (O_3_) forms through interactions with compounds like CO and NO_x_. Particulate matter (PM) air pollution includes various substances from traffic, industry, construction, fires, and waste incineration. PM is categorized by size: coarse PM (PM_10_), fine PM (PM_2.5_), and ultrafine PM (PM_0.1_) [2,3,4].

The Lancet Commission on pollution and health emphasized that deteriorated air quality stands as the primary environmental factor contributing to global disease and premature mortality. Diseases resulting from air pollution led to an estimated nine million premature deaths in 2015, surpassing the combined mortality of acquired immunodeficiency syndrome (AIDS), tuberculosis, and malaria by approximately threefold [5]. The primary contributor is ambient air pollution, which reduces the global average life expectancy by about 2.9 years, exceeding the impact of conventional health risk factors such as tobacco smoking (2.2 years) [6]. The WHO reports that up to 12.6 million global deaths in 2012 were linked to unhealthy environments [7,8]. Recent assessments indicate that in 2020 alone, nine million premature deaths worldwide were associated with air pollution in the form of PM_2.5_ [9,10]. 

While previous studies primarily focused on global or regional air pollution-related health effects, limited attention has been paid to comprehensively assessing the burden of disease in specific countries arising from air pollution. In the case of Afghanistan, a country navigating the challenges of post-conflict recovery and rapid urbanization, the health effects of air pollution emerge as a critical concern [11]. Afghanistan ranks prominently among the most air-polluted nations globally, with elevated concentrations of PM and other air pollutants posing significant environmental and public health challenges. In 2019, Afghanistan held the fourth position globally in the Air Quality Index (AQI) assessment of PM_2.5_ levels, indicating a notable standing among countries experiencing elevated pollution levels. This ranking positioned Afghanistan just behind Mongolia, Pakistan, and Bangladesh [12]. Exposure patterns in Afghanistan vary between outdoor and indoor sources. Urban areas with a high population density and increased industrialization experience elevated ambient air pollution, while rural areas, which rely on traditional cooking methods, contribute to indoor pollution that particularly affects vulnerable populations like women and children [13,14,15]. This analysis aims to shed light on the specific health effects experienced by the Afghan population due to exposure to air pollution. Understanding air pollution-related health impacts in Afghanistan is crucial for developing targeted interventions that align with the nation’s specific challenges and needs. To achieve this objective, recent data from the Global Burden of Disease (GBD) Study 2019 obtained from the Institute for Health Metrics and Evaluation were analyzed. Specific metrics (i.e., disability-adjusted life-years (DALYs), years of life lost (YLLs), years lived with disability (YLDs), and deaths) attributed to air pollution in Afghanistan were taken into consideration to characterize the burden of disease due to air pollution in Afghanistan.

## 2. Methods

### 2.1. Data Source

This analysis made use of data derived from the GBD Study 2019, providing an extensive evaluation of epidemiological variables like incidence, prevalence, deaths, years lived with disability (YLDs), years of life lost (YLLs), and disability-adjusted life years (DALYs) across 369 diseases and injuries, 286 mortality causes, and 87 risk factors spanning 204 countries and regions [16,17]. Pertinent information regarding the burden of disease in Afghanistan attributed to air pollution was extracted from the GBD Results tool available on the Institute for Health Metrics and Evaluation website (https://vizhub.healthdata.org/gbd-compare// accessed on 2 February 2024). For a more in-depth understanding of data sources, statistical analyses, and modeling procedures, readers are referred to the published works of the authors of the GBD 2019 Study [16,17].

### 2.2. Main Input Data

The calculation of DALYs in the GBD Study involved an examination of fundamental epidemiological data. Information from reviews, subsequent meta-analyses, and publicly available sources underwent rigorous statistical processing. Estimates, contingent on the quantity and quality of data, were primarily grounded in country-specific sources or derived from prediction models that considered incomplete or qualitatively insufficient data.

### 2.3. Measures

The outcomes of the GBD Study 2019 for Afghanistan are expressed in terms of deaths, YLDs, YLLs, and DALYs. DALYs consist of two essential components, YLLs and YLDs, serving as a metric for lost healthy life years. In the context of burden of disease studies, “disability” refers to any quantifiable (percentage) deviation from optimal health status. The mortality component of YLLs is calculated based on the number of deceased individuals (stratified by age, sex, and cause of death) and a globally standardized life expectancy at birth. The morbidity component of YLDs arises from the prevalence (stratified by age and sex) of the health-impairing condition under scrutiny and disability weights, standardized for all health states considered in the GBD Study 2019. These weights gauge the impact of diseases and injuries on health, ranging from 0 (complete health) to 1 (a state akin to death). The cumulative sum of YLLs and YLDs constitutes the DALYs [18].

### 2.4. Air Pollution—Level 2 Risk

Air pollution encompasses ambient particulate matter pollution (PM_2.5_), household air pollution from the use of solid fuels for cooking, and ambient ozone (O_3_) pollution [16]. The estimation of exposure to household air pollution arising from solid fuels involves an assessment based on two key parameters: the percentage of individuals utilizing solid cooking fuels and the level of exposure to PM_2.5_ among these individuals. Solid fuels encompass wood, coal/charcoal, dung, and agricultural residues. All presented results incorporate 95% uncertainty intervals (95% UI), which, similar to confidence intervals, encapsulate uncertainties related to estimation and also encompass uncertainties originating from various sources, such as modeling uncertainties.

## 3. Results

### 3.1. Air Pollution and All-Cause Measures

As shown in Table 1, the assessment of air pollution in Afghanistan in 2019 highlights a significant impact on various health measures. The estimated number of deaths attributed to air pollution amounted to 37,033, constituting 14.72% of all deaths. The overall DALY burden reached 1,849,170, making up 10.80% of Afghanistan’s total DALYs. YLDs were estimated at 76,858, representing 2.07% of total YLDs. YLLs due to air pollution were estimated at 1,772,311, comprising 13.23% of total YLLs.

### 3.2. Air Pollution and Measures for Communicable, Maternal, Neonatal, and Nutritional Diseases (CMNNDs) and Non-Communicable Diseases (NCDs)

Moreover, referring to Table 2, the impact of air pollution on CMNNDs and NCDs is presented in detail. Deaths attributed to CMNNDs resulting from air pollution were estimated at 13,459, constituting 19.21% of total CMNND-related deaths. NCDs contributed to 23,573 deaths, representing 17.87% of overall NCD-related mortality. DALYs associated with NCDs reached 772,784, comprising 10.19% of total DALYs attributed to NCDs. In the case of CMNNDs, the DALY burden was estimated at 1,076,385, constituting 17.84% of total DALYs assigned to CMNNDs. YLDs due to NCDs were estimated at 74,667, accounting for 3.10% of total YLDs attributed to NCDs. CMNNDs contributed to a smaller YLD burden of 2191 (0.33% of total YLDs related to CMNNDs). YLLs due to CMNNDs were estimated at 1,074,194, representing 20.04% of total YLLs assigned to CMNNDs. For NCDs, there were 698,117 YLLs, constituting 13.56% of total YLLs attributed to NCDs.

### 3.3. Air Pollution and Age-Specific Measures

**Figure 1:**
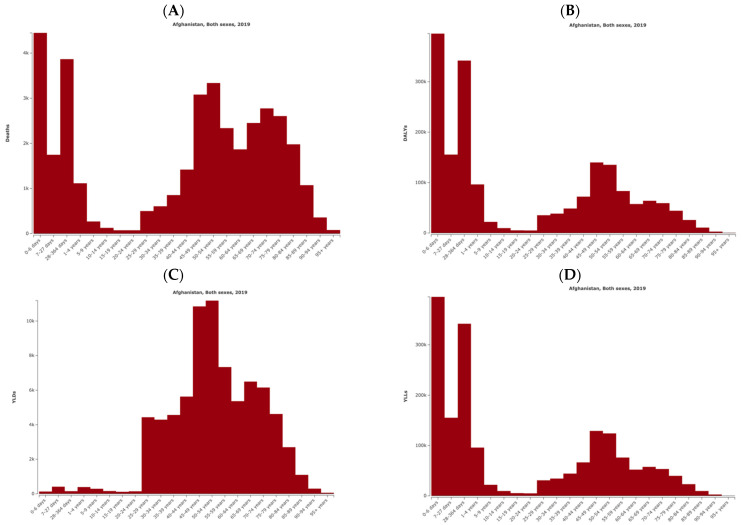
Numbers of deaths (**A**), disability-adjusted life-years (DALYs) (**B**), years lived with disability (YLDs) (**C**), and years of life lost (YLLs) (**D**) attributed to air pollution in Afghanistan for different age categories for the year 2019.

Figure 1 displays distinct patterns of mortality and disability across different age categories, emphasizing the differential health effects of air pollution on various age groups. For deaths, a bimodal distribution was observed, with two prominent peaks. The first peak was identified in the age category of 0 to 4 years, underscoring the heightened vulnerability of infants and young children to the adverse consequences of air pollution. The second peak was centered around the ages 40 to 84, indicating a significant impact on the older population. Intriguingly, the highest number of deaths was noted in the earliest days of life, specifically in the 0–6 day age category, highlighting the acute susceptibility of neonates to air pollution-related health risks. A parallel pattern was discerned in DALYs and YLLs, with a comparable bimodal distribution mirroring the peaks observed in the mortality data. Regarding the age distribution of YLDs, the highest numbers were observed in middle-aged categories, specifically around 45–49 years. 

### 3.4. Air Pollution and Cause-Related Measures

**Figure 2:**
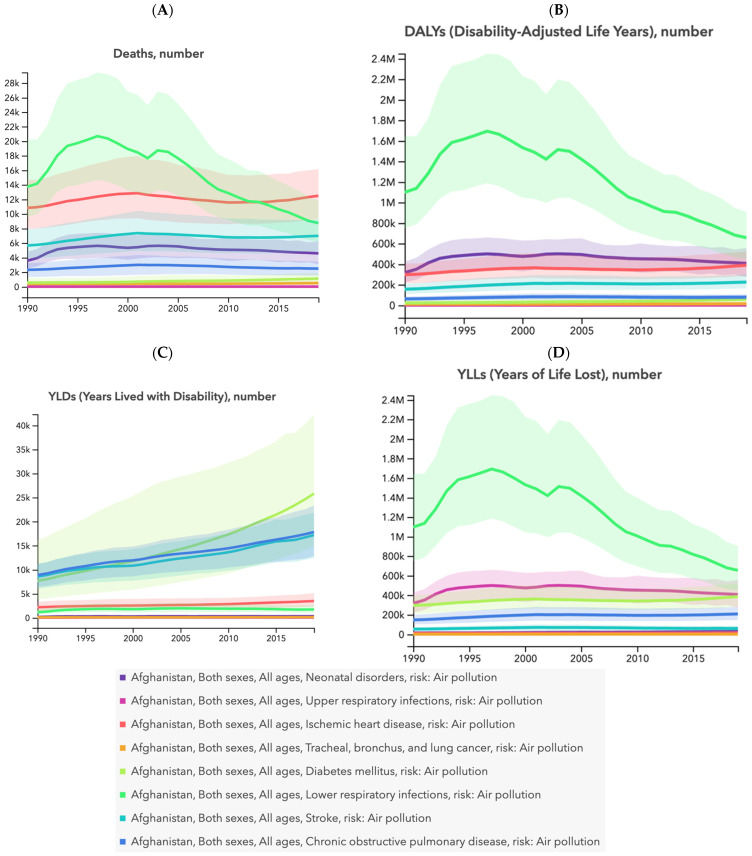
Time trend (1990 to 2019) for numbers (including uncertainty interval) of deaths (**A**), DALYs (**B**), YLDs (**C**), and YLLs (**D**) caused by diabetes mellitus; neonatal disorders; tracheal, bronchus, and lung cancer; upper respiratory infections; ischemic heart disease; lower respiratory infections; chronic obstructive pulmonary disease; and stroke attributed to air pollution in Afghanistan.

**Figure 3:**
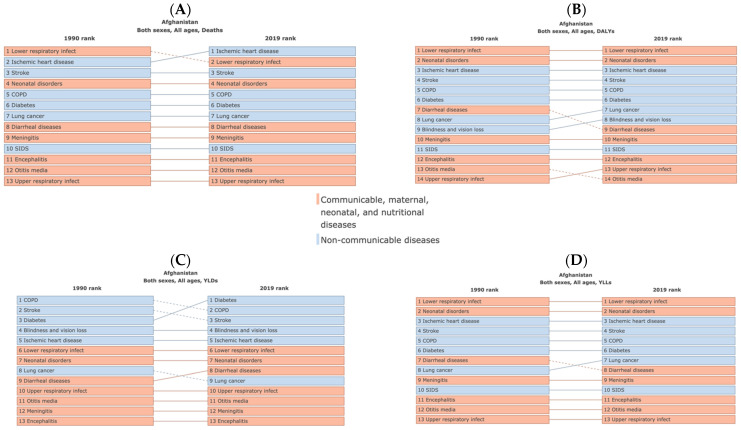
The leading causes for numbers of deaths (**A**), disability-adjusted life-years (DALYs) (**B**), years lived with disability (YLDs) (**C**), and years of life lost (YLLs) (**D**) attributed to air pollution in Afghanistan comparing the years 1990 and 2019.

Lower respiratory infections, neonatal disorders, ischemic heart disease, stroke, chronic obstructive pulmonary disease, lung cancer, and diabetes mellitus emerged as the predominant causes of both mortality and disease burden associated with air pollution in Afghanistan from 1990 to 2019 (Figure 2 and Figure 3). These specific health conditions were identified as major contributors to the number of deaths, DALYs, YLDs, and YLLs during the specified period. 

### 3.5. Ranking of Risk Factors

**Figure 4:**
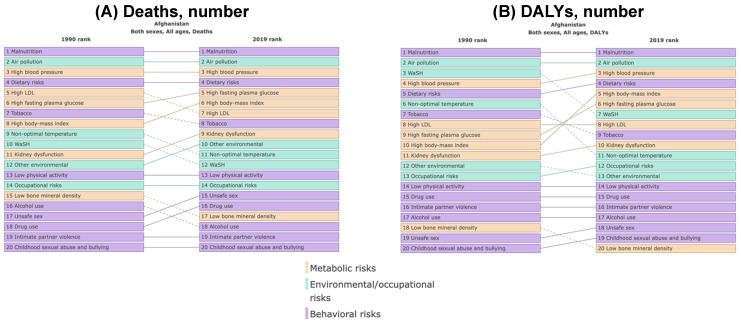

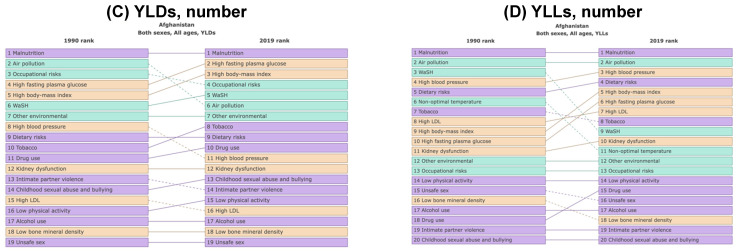
The most important risks for numbers of deaths (**A**), disability-adjusted life-years (DALYs) (**B**), years lived with disability (YLDs) (**C**), and years of life lost (YLLs) (**D**) in Afghanistan comparing the years 1990 and 2019.

Comparative assessments between 1990 and 2019 in Afghanistan shed light on the pivotal role of air pollution among the leading risk factors contributing to the burden of disease (Figure 4). Across both years, air pollution was consistently prominently ranked as a risk factor, closely competing with other significant contributors like malnutrition, high blood pressure, and dietary risks. Notably, in terms of the number of deaths, air pollution held the second position after malnutrition in 1990 and maintained this ranking in 2019. 

### 3.6. Air Pollution and Country-Specific Measures

**Figure 5:**
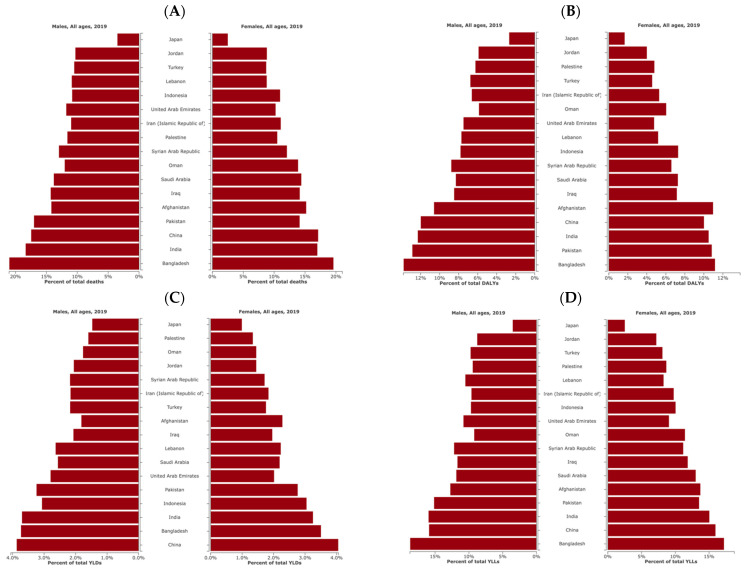
Percentages of deaths (**A**), disability-adjusted life-years (DALYs) (**B**), years lived with disability (YLDs) (**C**), and years of life lost (YLLs) (**D**) attributed to air pollution for males and females in selected countries for the year 2019.

Figure 5 illustrates percentages of health measures attributed to air pollution in Afghanistan in 2019, revealing a high-ranking position comparable to other selected countries known for a significant burden of disease due to air pollution, including China, India, Pakistan, and Bangladesh.

## 4. Discussion

The evaluation of air pollution in Afghanistan in 2019 unveiled a substantial impact on various health measures, highlighting the interplay between environmental factors and the national disease burden. The estimated 37,033 deaths attributed to air pollution, constituting 14.72% of the total, underscores a significant contribution to overall mortality. The burden of disease, quantified by DALYs, amounted to 1,849,170, representing 10.80% of Afghanistan’s total DALYs. This comprehensive metric encapsulates not only mortality, but also YLDs and YLLs, providing a nuanced understanding of the diverse impacts of air pollution exposure. Deaths attributed to CMNNDs represent a substantial proportion of total CMNND-related deaths, revealing distinctive age-specific patterns in mortality, with heightened vulnerability observed in neonates and the elderly. This analysis provided a nuanced understanding of specific health impacts, revealing distinctive age-specific patterns in mortality and other measures, with heightened vulnerability observed in neonates and the elderly. Respiratory diseases, cardiovascular conditions, and other NCDs emerged as predominant causes of death, DALYs, YLDs, and YLLs associated with air pollution in Afghanistan from 1990 to 2019. These findings draw attention to the consistent role of air pollution as a major health risk factor, persisting over decades despite fluctuations in exposure due to various environmental and societal changes. The continuous prominence of air pollution among the top contributors to deaths, DALYs, YLDs, and YLLs positions it alongside established health risk factors such as malnutrition, high blood pressure, and dietary risks. This continuity emphasizes the urgent need for comprehensive strategies that address environmental exposures in conjunction with established health risk factors to effectively mitigate the overall burden of disease in Afghanistan.

According to WHO estimates, deaths due to environmental risks constitute 26% of all deaths in Afghanistan, with household air pollution being the single most important environmental health risk factor [19]. The capital and largest city of Afghanistan, Kabul, recorded a PM_2.5_ reading of 58.8 μg/m³ in 2019 (mean annual value), ranking it as the 70th most polluted city globally [12]. Furthermore, the WHO estimated that household air pollution causes over 27,000 deaths per year, whereas ambient (outdoor) air pollution causes over 11,000 deaths in Afghanistan annually. Women and children are at particular risk of exposure to household air pollution, as they stay at home more than men [19]. Although there is a need for further research, epidemiological studies suggest that exposure to household air pollution contributes to low birth weight, stunting, and pre-term birth [20]. The primary sources of ambient air pollution in Afghanistan encompass motor vehicles, agricultural burning, and industrial activities. A primary natural source includes mineral dust generated through soil resuspension, predominantly originating from arid and semiarid soils [21,22]. Factors such as environmental degradation, inadequate infrastructure, accelerated urbanization, transportation emissions, industrial activities, and reliance on solid fuels collectively contribute to elevated levels of air pollutants. Distinct exposure patterns arise from both outdoor and indoor sources, with urban areas witnessing heightened ambient air pollution due to high population density and intensified industrialization. In contrast, rural regions relying on traditional cooking methods contribute to indoor pollution. Although the present study did not explicitly delve into the spatial distribution of air pollutants, it is important to acknowledge the importance of incorporating such assessments when evaluating health burdens. Importantly, spatial distribution can significantly differ between rural and urban areas, adding a layer of complexity to our understanding of air quality and its potential health effects. Urban areas often exhibit concentrated sources of pollution, resulting in localized high concentrations, while rural areas may experience more dispersed sources with varying levels of exposure. Recognizing these distinct patterns is essential for tailoring interventions and policies to address the specific challenges faced by diverse populations [23]. Further, it is crucial to contextualize these findings within the broader landscape of developing or low-income countries in Asia. Findings indicate that Afghanistan occupied a prominent position in the burden of disease attributable to air pollution compared to countries recognized for facing significant health challenges related to air pollution, specifically China, India, Pakistan, and Bangladesh. Across China, India, Pakistan, Bangladesh, and Afghanistan, sources of air pollution exhibit notable variations reflecting diverse socio-economic contexts and developmental stages. China faces emissions from extensive industrial infrastructure and coal combustion [24]. India encounters challenges from urbanization-driven transport-related emissions and industrial activities [25]. Pakistan deals with transportation pollution, industrial emissions, and agricultural practices [26]. Bangladesh copes with growing industrialization, increased traffic, and traditional biomass burning [27,28,29]. However, despite the imperative need for comprehensive investigations into Afghanistan’s specific air pollution sources, pathways, and health impacts, challenges such as limited monitoring infrastructure and data gaps pose impediments to scientific investigation [12,13,14,15].

Afghanistan’s air pollution crisis demands attention and strategic interventions. Although geopolitical challenges may hinder large-scale improvements, targeted initiatives, such as phasing out high-pollution vehicles and raising public awareness about the dangers of burning hazardous materials, can contribute to reducing pollution levels and protecting public health, especially that of vulnerable populations such as women and children. Mitigation efforts should prioritize source reduction, targeting prevalent contributors like motor vehicles, agricultural burning, and industrial activities. Urban planning interventions and infrastructure development can alleviate ambient air pollution, particularly in densely populated urban areas. In rural settings, promoting cleaner cooking technologies is pivotal to mitigating indoor pollution [12,13,14,15]. A recent study underscores the importance of phasing out fossil fuels, indicating that approximately 5.13 million excess deaths per year globally can be linked to ambient air pollution (including PM_2.5_ and O_3_) resulting from fossil fuel use. This suggests potential prevention of these deaths by transitioning to clean, renewable energy sources. This represents approximately 82% of the maximum number of air pollution-related deaths that could be avoided by controlling all human-made emissions [10]. 

## 5. Conclusions

Taken together, the present study’s analysis relied on data from the recent GBD Study 2019, which provided a solid basis for assessing the impact of air pollution in Afghanistan. Using diverse health metrics, this study’s findings offer nuanced insights into the health effects of air pollution, focusing specifically on the country’s challenges, age-specific patterns, and diseases linked to air pollution. However, limitations included data constraints from the GBD Study 2019, challenges in attributing health outcomes to specific sources, and gaps in monitoring infrastructure. External factors like seasonal variations and geopolitical challenges may not have been fully captured. Moreover, it is important to acknowledge that results regarding the influence of air pollution and malnutrition on health during the civil war may have been affected by factors such as migration. Despite these limitations, this study contributes valuable insights regarding evidence-based strategies to address air pollution’s health impacts in Afghanistan. In conclusion, this analysis highlights the urgent need for interventions to mitigate the public health impact of air pollution in Afghanistan. 

## Figures and Tables

**Table 1:** Deaths, disability-adjusted life-years (DALYs), years lived with disability (YLDs), and years of life lost (YLLs) attributed to air pollution in Afghanistan for the year 2019.

Measure	Metric	Value	UI Upper	UI Lower
Deaths	Number	37,033	45,042	29,774
	Percent	14.72	16.56	12.93
	Rate	96.75	117.67	77.78
DALYs	Number	1,849,170	2,250,663	1,485,560
	Percent	10.80	12.67	9.08
	Rate	4830.95	5879.85	3881.02
YLDs	Number	76,858	100,685	55,152
	Percent	2.07	2.55	1.68
	Rate	200.79	263.04	144.09
YLLs	Number	1,772,311	2,167,243	1,410,827
	Percent	13.23	15.28	11.27
	Rate	4630.16	5661.92	3685.79

Age-standardized rate per 100,000. UI: 95% uncertainty interval.

**Table 2:** Deaths, disability-adjusted life-years (DALYs), years lived with disability (YLDs), and years of life lost (YLLs) due to communicable, maternal, neonatal, and nutritional diseases (CMNNDs) and non-communicable diseases (NCDs) attributed to air pollution in Afghanistan for the year 2019.

Measure	Cause	Metric	Value	UI Upper	UI Lower
Deaths	CMNNDs	Number	13,459	17,335	10,118
		Percent	19.21	23.02	15.42
		Rate	35.16	45.29	26.43
	NCDs	Number	23,573	29,828	18,157
		Percent	17.87	20.72	15.35
		Rate	61.59	77.93	47.44
DALYs	CMNNDs	Number	1,076,385	1,403,066	793,828
		Percent	17.84	21.36	14.32
		Rate	2812.06	3665.51	2073.87
	NCDs	Number	772,784	981,498	597,716
		Percent	10.19	12.62	8.13
		Rate	2018.90	2564.16	1561.53
YLDs	CMNNDs	Number	2191	3152	1448
		Percent	0.33	0.42	0.25
		Rate	5.73	8.23	3.78
	NCDs	Number	74,667	98,168	53,437
		Percent	3.10	3.81	2.50
		Rate	195.07	256.47	139.61
YLLs	CMNNDs	Number	1,074,194	1,399,321	791,590
		Percent	20.04	23.98	16.08
		Rate	2806.33	3655.73	2068.03
	NCDs	Number	698,117	906,658	525,404
		Percent	13.56	17.31	10.55
		Rate	1823.83	2368.64	1372.62

Age-standardized rate per 100,000. UI: 95% uncertainty interval.

## Data Availability

Data are contained within the article.

## References

[1] Air Pollution. https://www.who.int/health-topics/air-pollution#tab=tab_1.

[2] Münzel T., Hahad O., Daiber A., Lelieveld J. (2021). Luftverschmutzung und Herz-Kreislauf-Erkrankungen. Herz.

[3] Hahad O., Kuntic M., Frenis K., Chowdhury S., Lelieveld J., Lieb K., Daiber A., Munzel T. (2021). Physical Activity in Polluted Air-Net Benefit or Harm to Cardiovascular Health? A Comprehensive Review. Antioxidants.

[4] Hahad O., Lelieveld J., Birklein F., Lieb K., Daiber A., Munzel T. (2020). Ambient Air Pollution Increases the Risk of Cerebrovascular and Neuropsychiatric Disorders through Induction of Inflammation and Oxidative Stress. Int. J. Mol. Sci..

[5] Landrigan P.J., Fuller R., Acosta N.J.R., Adeyi O., Arnold R., Basu N.N., Balde A.B., Bertollini R., Bose-O’Reilly S., Boufford J.I. (2018). The Lancet Commission on pollution and health. Lancet.

[6] Lelieveld J., Pozzer A., Poschl U., Fnais M., Haines A., Munzel T. (2020). Loss of life expectancy from air pollution compared to other risk factors: A worldwide perspective. Cardiovasc. Res..

[7] Ambient Air Pollution: A Global Assessment of Exposure and Burden of Disease. http://apps.who.int/iris/bitstream/10665/250141/1/9789241511353-eng.pdf?ua=1.

[8] Preventing Disease through Healthy Environments. https://www.who.int/quantifying_ehimpacts/publications/preventingdisease.pdf.

[9] Lelieveld J., Klingmuller K., Pozzer A., Poschl U., Fnais M., Daiber A., Munzel T. (2019). Cardiovascular disease burden from ambient air pollution in Europe reassessed using novel hazard ratio functions. Eur. Heart J..

[10] Lelieveld J., Haines A., Burnett R., Tonne C., Klingmuller K., Munzel T., Pozzer A. (2023). Air pollution deaths attributable to fossil fuels: Observational and modelling study. BMJ.

[11] Shah J., Essar M.Y., Qaderi S., Rackimuthu S., Nawaz F.A., Qaderi F., Shah A. (2022). Respiratory health and critical care concerns in Afghanistan. Lancet Respir. Med..

[12] Air Quality in Afghanistan Air Quality Index (AQI) and PM2.5 Air Pollution in Afghanistan. https://www.iqair.com/afghanistan.

[13] Waseq W.M. (2020). The Impact of Air Pollution on Human Health and Environment with Mitigation Measures to Reduce Air Pollution in Kabul, Afghanistan. Int. J. Healthc. Sci..

[14] Akbar K., Khaksar T.M. (2020). Impact of Air Pollution on Reproductive Health in Afghanistan. J. Adv. Pharm. Sci. Res..

[15] Mehrad A.T. (2020). Causes of Air Pollution in Kabul and its Effects on Health. Indian J. Ecol..

[16] GBD 2019 Risk Factors Collaborators (2020). Global burden of 87 risk factors in 204 countries and territories, 1990–2019: A systematic analysis for the Global Burden of Disease Study 2019. Lancet.

[17] GBD 2019 Diseases and Injuries Collaborators (2020). Global burden of 369 diseases and injuries in 204 countries and territories, 1990–2019: A systematic analysis for the Global Burden of Disease Study 2019. Lancet.

[18] Plass D., Vos T., Hornberg C., Scheidt-Nave C., Zeeb H., Kramer A. (2014). Trends in disease burden in Germany: Results, implications and limitations of the Global Burden of Disease study. Dtsch. Arztebl. Int..

[19] Afghanistan Environmental Health. https://www.emro.who.int/afg/programmes/eh.html#.

[20] Kaali S., Jack D.W., Mujtaba M.N., Chillrud S.N., Ae-Ngibise K.A., Kinney P.L., Boamah Kaali E., Gennings C., Colicino E., Osei M. (2023). Identifying sensitive windows of prenatal household air pollution on birth weight and infant pneumonia risk to inform future interventions. Environ. Int..

[21] Gasping for Air in Kabul. https://www.unep.org/news-and-stories/story/gasping-air-kabul.

[22] Lalonde J.D., Bradley M. 11 Years of Air Quality Monitoring in Afghanistan. https://military-medicine.com/article/3163-11-years-of-air-quality-monitoring-in-afghanistan.html.

[23] Han W., Li Z., Guo J., Su T., Chen T., Wei J., Cribb M. (2020). The Urban–Rural Heterogeneity of Air Pollution in 35 Metropolitan Regions across China. Remote Sens..

[24] Rohde R.A., Muller R.A. (2015). Air Pollution in China: Mapping of Concentrations and Sources. PLoS ONE.

[25] Guttikunda S.K., Goel R., Pant P. (2014). Nature of air pollution, emission sources, and management in the Indian cities. Atmos. Environ..

[26] Air Quality Index (AQI) and PM2.5 Air Pollution in Pakistan. https://www.iqair.com/us/pakistan.

[27] Khandker S., Mohiuddin A.S.M., Ahmad S.A., McGushin A., Abelsohn A. (2023). Air Pollution in Bangladesh and Its Consequences. Res. Sq..

[28] Begum B.A., Biswas S.K., Hopke P.K. (2011). Key issues in controlling air pollutants in Dhaka, Bangladesh. Atmos. Environ..

[29] Begum B.A., Hopke P.K., Markwitz A. (2013). Air pollution by fine particulate matter in Bangladesh. Atmos. Pollut. Res..

